# Genetic Prioritization, Therapeutic Repositioning and Cross-Disease Comparisons Reveal Inflammatory Targets Tractable for Kidney Stone Disease

**DOI:** 10.3389/fimmu.2021.687291

**Published:** 2021-08-20

**Authors:** Hai Fang, Lulu Jiang

**Affiliations:** ^1^Shanghai Institute of Hematology, State Key Laboratory of Medical Genomics, National Research Centre for Translational Medicine at Shanghai, Ruijin Hospital Affiliated to Shanghai Jiao Tong University School of Medicine, Shanghai, China; ^2^Bristol Renal Unit, Translational Health Sciences, University of Bristol, Bristol, United Kingdom; ^3^Department of Physiology Anatomy and Genetics, University of Oxford, Oxford, United Kingdom

**Keywords:** kidney stones, genetic targets, inflammatory pathways, drug repurposing, inflammasome, NK-kB regulation

## Abstract

**Background:**

Formation of kidney stones resulting in urological disorders remains a major cause of morbidity in renal diseases and many others. Innate immunity, mainly inflammasome, has demonstrated a key role in the development of kidney stone disease (or “nephrolithiasis”), but a molecular rationale for therapeutic intervention targeting immunity is far from clear. We reason that identifying inflammatory gene networks underlying disease risk would inform immunotherapeutic targets for candidate drug discovery.

**Results:**

We generated an atlas of genetic target prioritization, with the top targets highly enriched for genes involved in the NF-kB regulation, including interaction neighbors of inflammasome genes. We identified a network of highly ranked and interconnecting genes that are of functional relevance to nephrolithiasis and mediate crosstalk between inflammatory pathways. Crosstalk genes can be utilized for therapeutic repositioning, as highlighted by identification of ulixertinib and losmapimod that are both under clinical investigation as inhibitors of inflammatory mediators. Finally, we performed cross-disease comparisons and druggable pocket predictions, identifying inflammatory targets that are specific to and tractable for nephrolithiasis.

**Conclusion:**

Genetic targets and candidate drugs, *in silico* identified in this study, provide the rich information of how to target innate immune pathways, with the potential of advancing immunotherapeutic strategies for nephrolithiasis.

## Introduction

Experimental Factor Ontology (1) describes kidney stone disease (or “nephrolithiasis”) as a urological disorder, characterized by the presence of calculi (approximately 80% calcium stones) in the pelvis of the kidney. It is estimated that about one in 10 of us will suffer from nephrolithiasis in our lifetime (2), and the prevalence continues to increase, partly explained by lack of physical activities and unhealthy dietary choices (3). Nephrolithiasis is also thought as a systemic disorder with high economic health burden (4) not only accounting for renal diseases but also linked to other diseases such as diabetes (5). Standard care, such as pharmacological treatments and dietary preventions to increase intakes of water and potassium, can only achieve modest therapeutic efficiency (6), and new therapeutic strategies are much needed in order to reduce the disease burden.

Our understanding of the mechanisms of stone formation has made significant progress in identifying key physiochemical events such as crystal nucleation, aggregation, and retentions [reviewed in (7)]. Accordingly, therapeutic strategies can be either prevention of crystal nucleation and aggregation, or protection from renal epithelial cell injury which can increase retention force between crystals and injured cells. High calcium oxalate crystals are toxic that can cause oxidative stress-mediated renal cell injury (8), activate p38 MAPK signaling leading to necrotic cell death (9), and induce inflammatory responses through the NLRP3 inflammasome activation (10). There is increasing evidence to support the importance of macrophage polarization and autophagy in stone formation and clearance. Macrophage polarization can be modulated in favor of stone treatment and prevention (11, 12): towards anti-inflammatory macrophages (M2) away from proinflammatory macrophages (M1). M1 can stimulate inflammatory responses to promote stone formation, whereas M2 can promote stone phagocytosis to prevent kidney injury. Another prospective strategy for preventing the disease is to enhance autophagic activity, likely through the inhibition of mTOR (13); mTOR signaling is essential for modulating metabolism and other fundamental cell processes (14).

In addition to cellular modulation, genetic basis of kidney stones also starts to surface. For example, genome-wide association study (GWAS) of nephrolithiasis has been recently reported, identifying genetic loci that are likely to affect genes involved in vitamin D metabolism and calcium-sensing receptor signaling (15). The gene assignment from genetic loci, however, was simply based on genomic proximity. Such assignment did not consider regulatory effects of non-coding loci on genes, which could lead to false negatives during target gene discovery. We already know that loci arising from GWAS in common disease are mostly non-coding. We also know that regulatory effects of noncoding loci on target genes may involve 3D chromatin structure and are likely to be cell-type specific. In other words, the assignment of target genes from non-coding loci requires supports from a range of cell-type-specific regulatory genomic datasets, including but not limited to long-range physical chromatin interactions (16) and genetic regulation of gene expression (17). In an attempt to address this issue (i.e., reducing false negatives), we have established a genetics-led approach that can maximize the informativeness of GWAS and regulatory immunogenomics to enhance the drug target prioritization (18). Our approach is particularly useful in prioritizing immunomodulatory targets, taking advantages of a large body of immunogenomic datasets that have been generated in a wide variety of immune cell types and states. Our attempt is motivated by the fact that drug targets with genetic support are twice as likely to be therapeutically valid as those without support (19, 20).

Targeting innate immunity is an increasingly appreciated immunotherapeutic (mainly anti-inflammatory) strategy for kidney stone disease (12). In this study, we sought to provide a molecular rationale for therapeutic intervention in nephrolithiasis, exploring genetic evidence arising from GWAS in nephrolithiasis and regulatory immunogenomic datasets. We performed genetic prioritization on a genome wide and identified a gene network responsible for crosstalk between pathways that are essential for inflammation. Based on pathway crosstalk genes, we also performed therapeutic repositioning and *in silico* predicted therapeutic targets and drug combinations. Further cross-disease comparisons with immune-mediated diseases allowed us to identify inflammatory target candidates that are specific to nephrolithiasis.

## Materials and Methods

### Genetic Prioritization at the Gene, Pathway, and Crosstalk Level

GWAS summary data in nephrolithiasis were obtained from previous studies (15, 21, 22) and analyzed using the Pi package (version 2.2.1), our recently established genetics-led target prioritization (18). In brief, GWAS SNPs (including SNPs in linkage disequilibrium) were used to define genes under genetic influence, including nearby genes (*nGene*) based on genomic proximity and organization (23), expression-associated genes (*eGene*) integrating eQTL datasets (24–26), and conformation genes (*cGene*) using promoter capture Hi-C datasets (27). These defined genes were then used to identify additional genes through exploiting high-quality gene interaction information from the STRING database (only with evidence codes “experiments” or “databases”) (28). This resulted in a total of 12,466 target genes prioritized, for which priority rating (scored 0–5) was visualized using Manhattan plot.

Individual pathways were prioritized based on enrichment analysis of top prioritized genes. Enrichment analysis was performed according to one-sided Fisher’s exact test, done so separately using KEGG pathways (29) and Reactome pathways (30). The enrichments were measured by Z-score, odds ratio (OR), and false discovery rate (FDR). The identification of crosstalk between pathways was achieved by searching for a subset of gene interactions (merged from KEGG pathways) so that the resulting pathway crosstalk contained highly ranked and interconnecting genes. The significance (*p*-value) of the identified crosstalk was estimated by a degree-preserving node permutation test (100 times). In addition to being visualized naturally as a gene network, the crosstalk was also illustrated as a pathway-centric network, with pathways as nodes and their estimated connections as edges. Only pathways significantly overrepresented in crosstalk genes were considered nodes. The edges were initially inferred if member genes were shared between pathways, and then filtered by identifying the minimum spanning tree using the igraph package (version 1.2.6) (31) to keep only edges found in the resulting tree. The thickness of edges was proportional to the number of member genes shared between two-endpoint pathways.

### Target Set Enrichment Analysis Using the Hallmark Gene Sets

As the nonredundant version of the MSigDB database, the hallmark gene sets capture the comprehensive but representative information on molecular pathways (including KEGG, Reactome, and many others) and biological knowledge about gene regulations and many others (32). We used the dnet package (version 1.1.7) (33) to perform rank-based target set enrichment analysis (conceptually similar to gene set enrichment analysis (34), quantifying the degree to which a hallmark gene set was enriched at the “leading prioritization” of the ranked gene list. The leading prioritization was defined as the left-most region, containing the core subset of the prioritized genes accounting for the enrichment signals. The significance was quantified using FDR and normalized enrichment score (NES).

### Definition of Inflammasome Genes, Nephrolithiasis Genes, and Their Interaction Neighbors

Inflammasome genes were defined using Gene Ontology (35), restricted to inflammasome-related terms including inflammasome complex (GO:0061702), regulation of NLRP3 inflammasome complex assembly (GO:1900225), NLRP3 inflammasome complex assembly (GO:0044546), and NLRP1 inflammasome complex assembly (GO:1904784). Known nephrolithiasis genes were sourced from gene annotations using Disease Ontology (36), restricted to a term nephrolithiasis (DOID:585). Interaction neighbors were identified based on the STRING database (28), restricted to high-quality gene interactions with evidence codes “experiments” or “databases”.

### Therapeutic Repositioning and Removal Analysis

Therapeutic repositioning was based on information on current therapeutics (including drugs, development phases, target genes, and disease indications) in the ChEMBL database which curates therapeutic information mainly sourced from ATC, ClinicalTrials.gov, DailyMed, and FDA (37). Given a disease indication, drugs with the maximum phase of development were selected for a target gene, given that selected target genes had well-defined mechanisms of action and explained the efficacy of drugs in disease. Selected target genes were also categorized into two groups: one for approved drug targets (that is, genes targeted by any approved drugs), and the other for phased drug targets (that is, genes targeted by non-approved phased drugs). One-sided Fisher’s exact test was used to evaluate the significance of pathway crosstalk genes that were enriched for approved drug targets and phased drug targets.

The effect of nodes on the network was evaluated using removal analysis, done so for individual nodes and nodes in combination. The nodes removed (either individually or in combination), if critical for the network, would result in a large fraction of nodes disconnected from the largest network component after node removal. Combinatorial removal analysis was carried out to select drug combination (i.e., optimal combination) maximizing the effect of removing nodes, that is, the largest fraction of disconnected nodes observed for a specific node combination removed. The effect of node removal was visualized using the ggupset package (version 0.3.0).

### Cross-Disease Comparisons Using the Supra-Hexagonal Map

Focusing on the pathway crosstalk genes identified in nephrolithiasis, the supraHex package (version 1.28.1) was used to compare prioritizations against immune-mediated diseases [sourced from (18)]. In brief, a supra-hexagonal map consisting of 19 hexagons was trained using the self-organizing learning algorithm [referred to (38) for details]. The diseases selected for comparisons covered a wide spectrum of autoinflammatory–autoimmune continuum, subdivided into: (i) autoinflammatory diseases, comprising Crohn’s disease and ulcerative colitis; (ii) autoimmune diseases, including multiple sclerosis, rheumatoid arthritis, systemic lupus erythematosus, and type 1 diabetes; and (iii) in-between mixed diseases, including ankylosing spondylitis and psoriasis.

The trained map was utilized for downstream analyses. Firstly, the map was used to visualize disease-specific prioritization profiles, with diseases organized onto a 2D square lattice in a manner that diseases with similar profiles were placed closer to each other. Secondly, the map was partitioned into four target gene clusters, each having similar prioritization patterns across diseases. Thirdly, the map was overlaid with druggable pocket data (binary) for estimating the probability of each hexagon containing druggable/tractable genes. A gene was defined to be tractable if its known protein structure(s) predicted to contain drug-like binding sites (that is, druggable pocket). The known protein structures were available from the PDB database (39), and druggable pockets predicted using the fpocket software (version 2.0) (40). The significance of pathway crosstalk genes enriched in pocket-containing genes was evaluated using one-sided Fisher’s exact test.

## Results

### Genetic Prioritization Highlights the Importance of the NF-kB Regulation in Kidney Stone Disease

**Figure 1:**
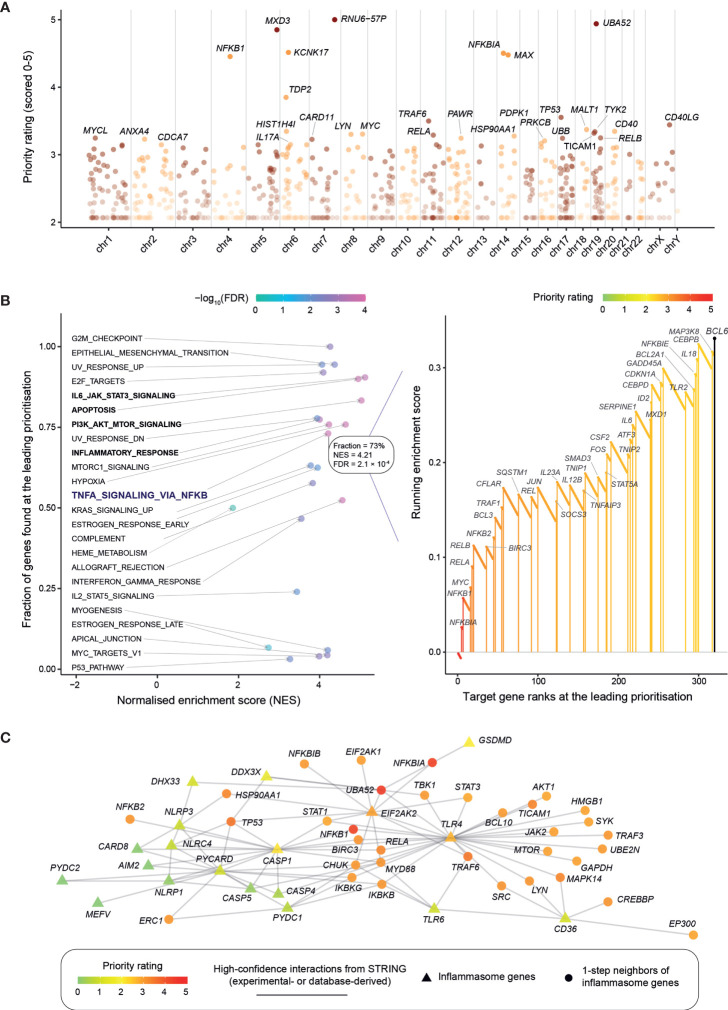
Genetic prioritization and characterization of target genes in kidney stone disease. **(A)** Overview of target gene prioritization. Manhattan plot illustrates priority rating (*y*-axis) for prioritized target genes (color-coded by chromosomes; *x*-axis), with top 30 genes named. **(B)** Target set enrichment analysis (TSEA) using the hallmark gene sets. *Left panel*: Scatter plot showing TSEA results, with each dot for a gene set and colored by FDR. *Right panel*: Illustration of the leading prioritization for the gene set “*TNFA_SIGNALING_VIA_NFKB*” (that is, genes regulated by NF-kB in response to TNF). The leading prioritization is defined as the left-most region ahead of the peak, as indicated by the dark blue bar. Genes found at the leading prioritization are indicated in vertical lines (also color-coded by priority rating). **(C)** Inflammasome. Interactions between inflammasome genes are visualized, together with their one-step (direct) neighbor genes (restricted to the top 1% prioritized genes). Gene nodes are colored by priority rating and shaped by whether or not functionally relevant to inflammasome. Gene interactions are sourced from the STRING database, while the relatedness to inflammasome obtained from annotations using Gene Ontology.

Using GWAS summary data in nephrolithiasis (15, 21, 22), we first employed our genetics-led target prioritization approach (18) to generate a prioritized list of >12,000 target genes (**Table S1**). Manhattan plot illustrates target genes ranked by priority rating (**Figure 1A**). The top-ranked genes are mostly involved in the NF-kB signaling, such as *NFKBIA* (top 5th), *NFKB1* (7th), *RELA* (19th), and *RELB* (21st), to name but just a few. Using hallmark gene sets (32), we then performed rank-based target set enrichment analysis (TSEA; **Figure 1B** and **Figure S1**), revealing that highly prioritized genes tend to be regulated by NF-kB in response to TNF, the key regulation in inflammation. Within this inflammatory regulation, 73% (38/52) of genes were found at the leading prioritization. TSEA also revealed the tendency of highly prioritized genes to be involved in inflammation-related events, including PI3K-AKT-mTOR signaling, IL6-JAK-STAT3 signaling, apoptosis by caspase activation, and inflammatory response (**Figure S1**).

Now we already know that inflammatory response can be induced by high calcium oxalate crystals, triggering the release of proinflammatory cytokines such as interleukin (IL)-1β and IL-18 through the inflammasome activation (41). These two proinflammatory cytokines, *IL1B* (349th) and *IL18* (303rd), were moderately prioritized. Interestingly, our genetic prioritization did not highly rate inflammasome genes (in other words, lacking genetic evidence; see **Table S2**), instead, highly rated were interaction neighbors of inflammasome genes. Among the top 1% of prioritized genes, there exists only one (*EIF2AK2*) out of 21 inflammasome genes (*p* = 0.19 on Fisher’s exact test); this is in sharp contrast to 36 interaction neighbors (out of 295 in total; *p* = 1.2 × 10^−29^), and these neighbors are mostly related to the NF-kB signaling (**Figure 1C**). For example, it is not the gene *GSDMD* (encoding gasdermin D) but its interaction gene *NFKBIA* that is highly rated. As part of inflammasome, *GSDMD* is well studied for its importance in programmed necrotic cell death or “pyroptosis” (42). Like inflammasome genes, we also observed similar findings for nephrolithiasis genes: very few of nephrolithiasis genes ranked at the top 1% (*p* = 0.09) versus many of their interaction neighbors highly rated (*p* = 7.9 × 10^−29^; **Figure S2** and **Table S3**). In summary, our genetic prioritization identifies the NF-kB regulation as a central mediator of inflammation that is likely essential for kidney stone disease.

### Pathway Prioritization Identifies Crosstalk Between Inflammatory Pathways in Kidney Stone Disease

**Figure 2:**
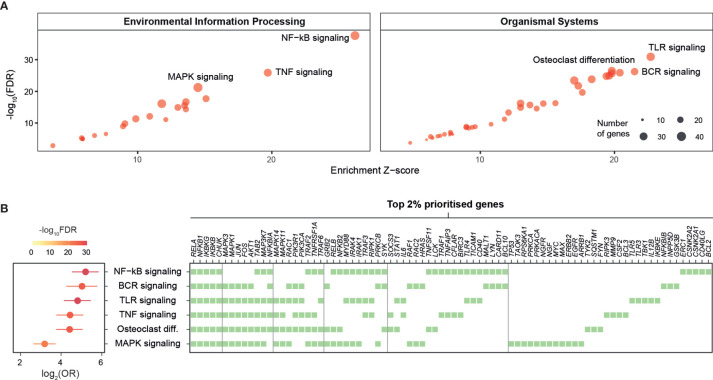
Prioritization of target pathways in kidney stone disease. Pathway-level prioritization was based on enrichment analysis of the top 2% prioritized genes using a collection of KEGG pathways, with the enrichment Z-score, the significance level (FDR), odds ratio (OR), and 95% confidence intervals estimated according to Fisher’s exact test (one-sided). BCR, B-cell receptor; MAPK, mitogen-activated protein kinase; TLR, toll-like receptor; TNF, tumor necrosis factor. **(A)** Scatter plots illustrating prioritized pathways, with the top 3 displayed separately for environmental information processing pathways (left) and organismal systems pathways (right). Each dot represents an individual pathway and is sized by the number of member genes thereof. **(B)** Forest plots of the top 6 prioritized pathways (left), with member genes illustrated using a heatmap (right) in which genes are shown at the top and ordered by frequency of occurrence (colored square).

**Figure 3:**
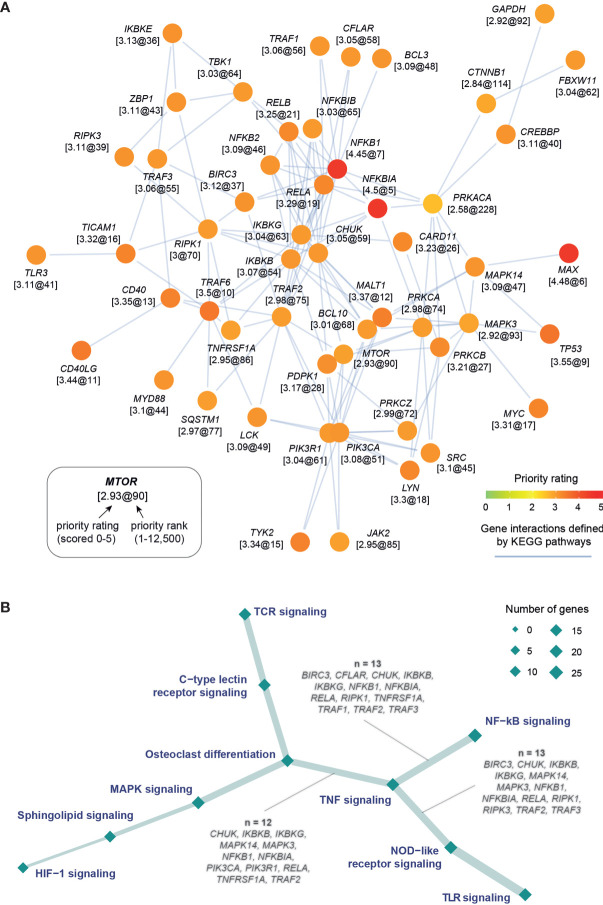
Target pathway crosstalk identified in kidney stone disease. **(A)** Gene-centric representation of the crosstalk, with nodes for genes and edges for interactions between nodes. Node are labelled by gene symbols along with the priority information (formatted as “*rating@rank*”) and colored by priority rating. This crosstalk was identified to contain highly prioritized interconnecting genes from a network of gene interactions (obtained by merging KEGG pathways). **(B)** Pathway-centric representation of the crosstalk, with nodes for pathways (sized by the number of member genes) and edges for connections (the thickness proportional to the number of genes shared between two-endpoint pathways). Also labeled in the edges are shared genes between the TNF signaling and the other endpoint as indicated.

To enhance the findings above on the importance of NF-kB signaling and further identify additional pathways that can also play a pivotal role in kidney stone disease, we next used the KEGG resource (29) to prioritize pathways based on enrichment analysis of highly prioritized genes. The top prioritized pathways include NF-kB, TNF, and TLR signalings (**Figure 2A**), and these pathways were consistently identified (**Figure S3**). Interestingly, commonly involved in these pathways are key players (*RELA*, *NFKB1*, *IKBKG*, *IKBKB*, and *CHUK*) of the NF-kB regulation (**Figure 2B**). We also used the Reactome resource (30) and obtained similar but more specific pathways, such as TRAF6- or TNFR1-induced NF-kB activation (**Figure S4**). To complement the knowledge obtained from individual pathway prioritizations, we next exploited interaction information merged from all KEGG pathways to identify a pathway crosstalk that contains highly ranked and interconnecting genes (*p*=3.2×10^−99^ on permutation test; **Figure 3A** and **Table S4**). Genes in this crosstalk include those previously reported to be of functional relevance to nephrolithiasis, such as protein kinase C (*PRKCA*, *PRKCB*, and *PRKCZ*) (43), markers (*CD40* and *TLR3*) (44), and autophagy (*MTOR* and *SQSTM1*) (13), thus validating our genetic prioritization at the gene and pathway level. Based on pathways significantly over-represented in the crosstalk (**Figure S5**), we further constructed crosstalk at the pathway level, with edges estimated by the extent to which member genes are shared between two endpoints (**Figure 3B**). This pathway-centric representation can be useful to identify points for simultaneously targeting multiple inflammatory pathways, for example, targeting NF-kB, TNF, and NOD-like signalings through their shared genes (*IKBKB*, *IKBKG*, *NFKB1*, *NFKBIA*, *RELA*, and *TRAF2*).

### Pathway Crosstalk Genes Inform Repositioning of Existing Therapeutics to Kidney Stone Disease

**Figure 4:**
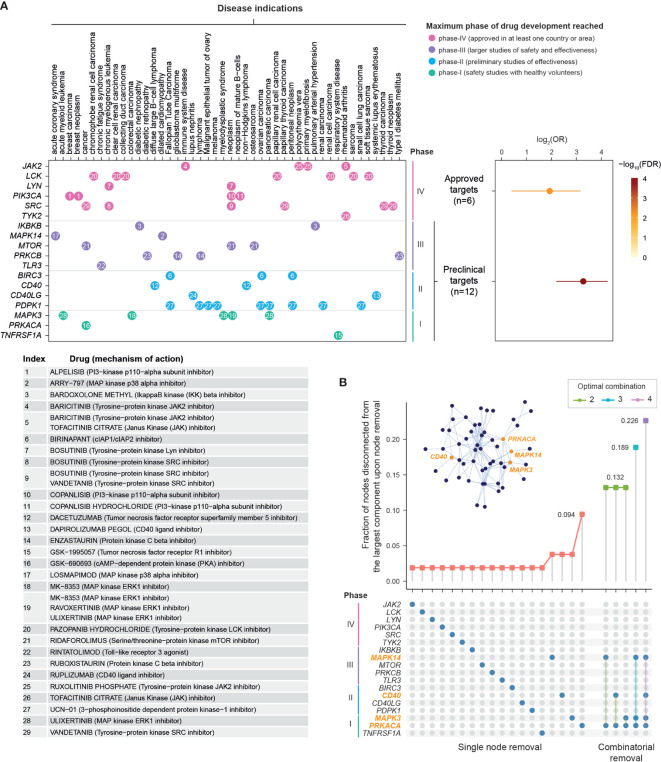
Drug repurposing analysis of pathway crosstalk genes. **(A)** Support from existing therapeutics. *Top-left panel*: dot plot showing 18 crosstalk genes (*y*-axis) that are currently targeted by approved (phase IV) and phase I/II/III drugs in disease indications (*x*-axis). Dots are colored by maximum phase of drug development reached and indexed in number. *Bottom-left panel*: Information on drugs and mechanism of action. *Top-right panel*: Forest plots of approved or phased drug targets enriched in crosstalk genes. The significance level (FDR), odds ratio (OR), and 95% confidence intervals (represented by lines) calculated according to Fisher’s exact test (one-sided). **(B)** Rational drug selection. It is based on effects of node removal on the crosstalk. Fraction of nodes disconnected from the largest remaining component (*y*-axis) is plotted against node removal (*x*-axis). The nodes removed are indicated by blue circles beneath. Notably, nodes removed in combination are indicated by linked blue circles. For each combinatorial removal, only the optimal combinations with the largest effect are shown (also color-coded). Inserted is the illustration of the crosstalk, with the same layout as shown in **Figure 3A** but only labeled for four nodes in optimal combinations.

Next, we explored the evidence supporting drug repurposing based on whether pathway crosstalk genes are targeted by approved or phased drugs (**Figure 4A** and **Table S5**). Using the well-curated information of existing therapeutics available in the ChEMBL database (37), we found the high degree of support from clinical evidence (approved drugs; FDR = 6.6 × 10^−3^), identifying six genes (*JAK2*, *LCK*, *LYN*, *PIK3CA*, *SRC*, and *TYK2*) for repurposing of licensed medications (that is, drugs already in clinical use). We also identified 12 preclinical (phased) drug targets, showing higher degree of support from preclinical evidence (FDR = 6.4 × 10^−8^). These include five genes (*IKBKB*, *MAPK14*, *MTOR*, *PRKCB*, and *TLR3*) targeted by clinical phase-III drugs, four phase-II drug target genes (*BIRC3*, *CD40*, *CD40LG*, and *PDPK1*), and three phase-I drug target genes (*MAPK3*, *PRKACA*, and *TNFRSF1A*).

We proceeded to reveal target-specific therapeutic potential by quantifying the tolerance of the pathway crosstalk to node removal of 18 approved and phased targets, done so individually and in combination (**Figure 4B**). The effect of node removal can be measured as the fraction of nodes disconnected from the largest remaining component after removal. Removing a node critical for the crosstalk would result in a large number of disconnected nodes. We found that the crosstalk was very robust to single node removal, with the maximum effect (~10% disconnected nodes) achieved by removing *PRKACA*. The robustness to single node removal motivated us to further examine the effect of combinatorial removal. Moreover, in order to achieve an adequate effect while minimizing adverse effects, it is logical to identify the smallest possible number of nodes in combination. For these two reasons, we removed between two to four nodes in combination to identify the optimal combination. We found that the *PRKACA* inhibitor could be combined with an agent inhibiting *MAPK14*, *MAPK3*, or *CD40* to achieve the same effect (~13%). With *PRKACA* at hand, removing another two nodes (*MAPK3* and *MAPK14*) was predicted to disconnect 18.9% of nodes; accordingly, both inhibitors of inflammatory mediators (that is, ulixertinib targeting *MAPK3* and losmapimod targeting *MAPK14*) were predicted to be the promising agents. The effect of three-node removal could be further increased to 22.6% with the addition of dacetuzumab, a monoclonal antibody targeting *CD40*.

### Cross-Disease Comparison Suggests Tractable Targets Specifically for Kidney Stone Disease

**Figure 5:**
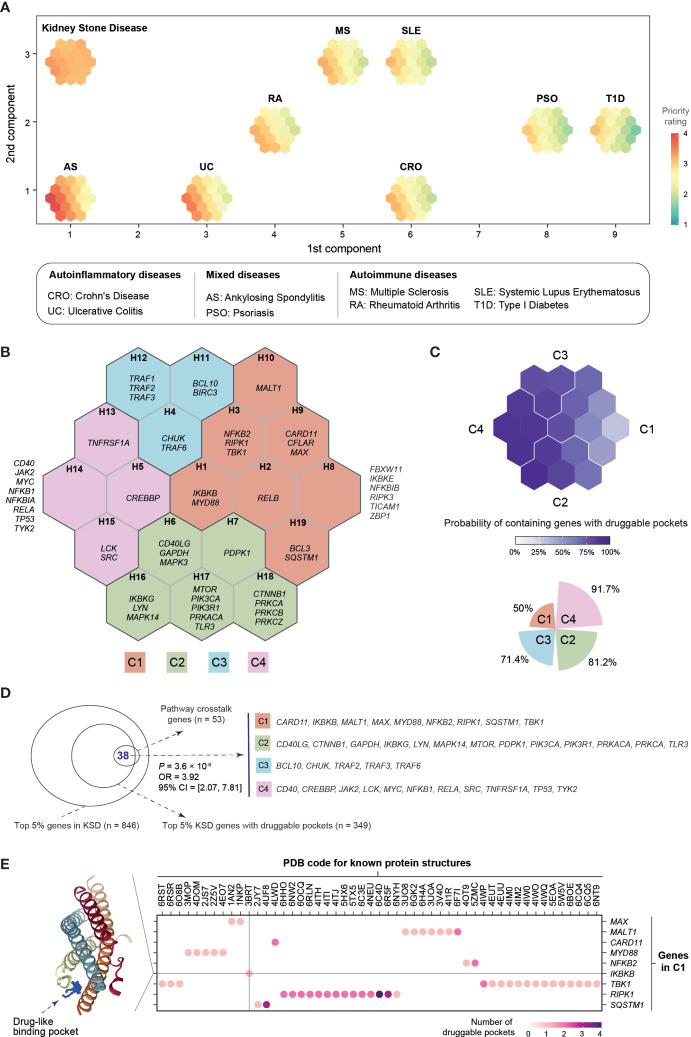
Cross-disease analysis of pathway crosstalk genes. The target prioritizations for eight immune-related disease traits are sourced from the Pi database. On the basis of degree of autoinflammation versus autoimmunity, these immune traits are grouped into polygenic autoinflammatory diseases (CRO and UC), polygenic autoimmune diseases (MS, RA, SLE and T1D), and mixed diseases having both (AS and PSO). **(A)** Comparisons using the supra-hexagonal map. This map was learned from the prioritization information of 53 crosstalk genes in kidney stone disease (KSD) and eight immune-related traits. Each map illustrates a trait-specific crosstalk gene prioritization profile. Across traits, genes with similar prioritization patterns are mapped onto the same or nearly position in the map. The outermost frame represents the landscape for the traits analyzed, from which geometric locations of traits delineate their relationships (by the similarity of prioritization profiles between traits). **(B)** Gene clusters. The map was divided into four target gene clusters (C1–C4) as color coded; each covering continuous hexagons. Hexagons are indexed and expanded outward (H1–H19). Also displayed are genes found in each hexagon. **(C)** Tractability. The map is color-coded by the probability of each hexagon containing tractable genes, with the percentage of tractable genes summarized for each cluster shown beneath (polar bar). A gene is defined as being tractable if predicted to contain a druggable pocket. **(D)** Venn diagram illustrating the enrichment for tractable genes. The significance level (P), odds ratio (OR), and 95% confidence intervals (CI) calculated using one-sided Fisher’s exact test. KSD, kidney stone disease. **(E)** Druggable pockets. Dot plot shows nine tractable genes in C1 (*y*-axis) and their PDB known protein structures (*x*-axis). Color-coded is the number of druggable pockets predicted in the structure. An example structure “3BRT” is visualized on the left, with a druggable pocket illustrated using 3D balls in blue.

Finally, focusing on pathway crosstalk genes, we compared prioritizations in kidney stone disease with regard to our previously published prioritizations in immune-mediated diseases (18). We found no or weak correlations of priority ratings in kidney stone disease with those in immune diseases (**Figure S6**), except for modest correlations observed for multiple sclerosis (Pearson’s correlation = 0.348, *p* = 1.1 × 10^−2^). To identify genes that are shared between diseases and are unique to a specific disease, we also employed a supra-hexagonal map (38) for cross-disease comparisons (**Figure 5A**). This identified four target clusters (C1–C4), each containing genes with similar prioritization patterns (**Figure 5B** and **Figure S7**; **Table S6**). Among these, the cluster C4 was highly rated in all diseases analyzed, displaying the highest tractability (**Figure 5C**); a tractable gene is defined to contain druggable pockets predicted from the known protein structure (39, 40). Notably, the cluster C1 was highly rated in kidney stone disease only and contained the relatively low proportion of tractable genes (**Figure 5C**), indicative of being underexplored. Overall, crosstalk genes were significantly enriched for druggable pockets (*p* = 3.6 × 10^−6^ on Fisher’s exact test; **Figure 5D**). We suggest that the pocket-containing genes that are prioritized highly and specifically in kidney stone disease (**Figure 5E**) are of particular interest for future validation.

## Discussion

Genetic evidence arising from human disease genomics can inform the discovery of therapeutic targets (45). Implementation of genetics-led drug target selection remains a prospective area for computational translational research; we have pioneered this effort [reviewed in (46)]. Our genetic prioritization utilizes previously published GWAS data of nephrolithiasis, but our findings convey the messages beyond what the original studies have revealed. We have prioritized genetic targets from an innate immunity perspective. It is worth noting that our approach is unique in respecting the omnigenic model of genetic architecture (47, 48). In essence, we have considered potential targets: not only (very few) core genes directly inferred from GWAS summary data and regulatory immunogenomic data but also (very huge) peripheral genes that are linked to core genes through gene connectivity. This might explain why previously reported nephrolithiasis genes (and inflammasome genes as well) are very limited in numbers. Consistent with this, we do not highly prioritize previously reported genes (lacking genetic evidence), instead, their interaction neighbors are highly rated. Interestingly, most of highly rated neighbors converge on the NF-kB regulation, a central mediator of inflammation.

Our genetic prioritization does not pick up cytokines that act on cells of adaptive immune responses (**Table S1**; with ranks greater than 1,000 out of >12,000 target genes); such cytokines include *IL2* and its receptors *IL2RA*, *IL2RB*, and *IL2RG* that mainly act on T cells, B cells, and NK cells; *IL3* and *IL3RA* on T cells and NK cells; *IL4* and *IL4R* on T helper cells, B cells, T cells, and macrophages; and *IL5* and *IL5RA* on T helper cells, B cells, and eosinophils. In other words, based on existing data there is lack of genetic evidence targeting adaptive immune pathways for nephrolithiasis. Given the possible role of adaptive immunity in disease, we anticipate that future availability of more immunogenomic datasets, particularly in adaptive immune cell types and states, may increase the chance of identifying the link with adaptive immunity.

Our identified pathway crosstalk contains several genes that have already been reported to be functionally relevant to nephrolithiasis. These include genes involved in autophagy, such as the gene *MTOR* responsible for autophagy defects and the gene *SQSTM1* encoding the autophagic substrate. The previous study has proposed that the deregulated MTOR signaling and the impairment in autophagy represent promising targets for suppressing kidney stone development (13). Our study suggests that autophagy-related genes likely act in a wider context and may interact with other genes, collectively forming as a cohesive network of inflammatory pathways (such as NF-kB, TNF, MAPK, TLR, and NOD-like signalings).

Genes in our identified pathway crosstalk are highly interconnected, and the nature of high interconnectivity makes it not so easy to be perturbed. In addition to removal analysis for individual nodes or nodes in combination, we have also performed “targeted attack” (49), an analysis that sequentially removes nodes that are preordered in a specific manner (**Figure S8**). Such successive node removal can be based on either node centrality (measured by degree or betweenness) or node priority (here priority rating). We found that the crosstalk was tolerant to targeted attack by node priority; it requires 40% nodes to be removed to achieve 50% nodes disconnected. Relatively, the crosstalk is vulnerable to targeted attack by node centrality; disconnecting 50% nodes only requires removing 15% nodes. Overall, the robustness to targeted attack suggests the difficulty in the design and implementation of therapeutic intervention in nephrolithiasis. Indeed, even combinatorial removal on any four nodes in the crosstalk altogether can maximally disconnect as low as 23% nodes (**Figure S9**). These results also suggest the necessity of the computational design of pharmaceutical agents acting on multiple targets to achieve efficiency in treating kidney stone disease.

In summary, our genetic targets provide the rich information of how to target innate immune pathways, with the potential of advancing immunotherapeutic strategies for nephrolithiasis. We anticipate that our pathway crosstalk-based analysis can be an inspiration for future studies to enhance the uptake of genetic prioritization and therapeutic repositioning by the scientific community and pharmaceutical companies working in kidney stones and beyond.

## Data Availability Statement

The data and source codes supporting our findings are made publicly available at https://23verse.github.io/kids.

## Author Contributions

LJ analyzed data, contributed to data interpretation, and wrote the manuscript. HF conceived and supervised the project, performed the analysis, and wrote and revised the manuscript. All authors contributed to the article and approved the submitted version.

## Funding

This research is funded by the Program for Professor of Special Appointment (Eastern Scholar) at Shanghai Institutions of Higher Learning (awarded to HF).

## Conflict of Interest

The authors declare that the research was conducted in the absence of any commercial or financial relationships that could be construed as a potential conflict of interest.

## Publisher’s Note

All claims expressed in this article are solely those of the authors and do not necessarily represent those of their affiliated organizations, or those of the publisher, the editors and the reviewers. Any product that may be evaluated in this article, or claim that may be made by its manufacturer, is not guaranteed or endorsed by the publisher.
