# Supplementary material for: Genetic Prioritization, Therapeutic Repositioning and Cross-Disease Comparisons Reveal Inflammatory Targets Tractable for Kidney Stone Disease
Source: Front Immunol. 2021 Aug 20;12:687291. doi: 10.3389/fimmu.2021.687291 (PMC8417698; doi:10.3389/fimmu.2021.687291)
Supplement: Supplementary file 1 [file DataSheet_1.pdf]

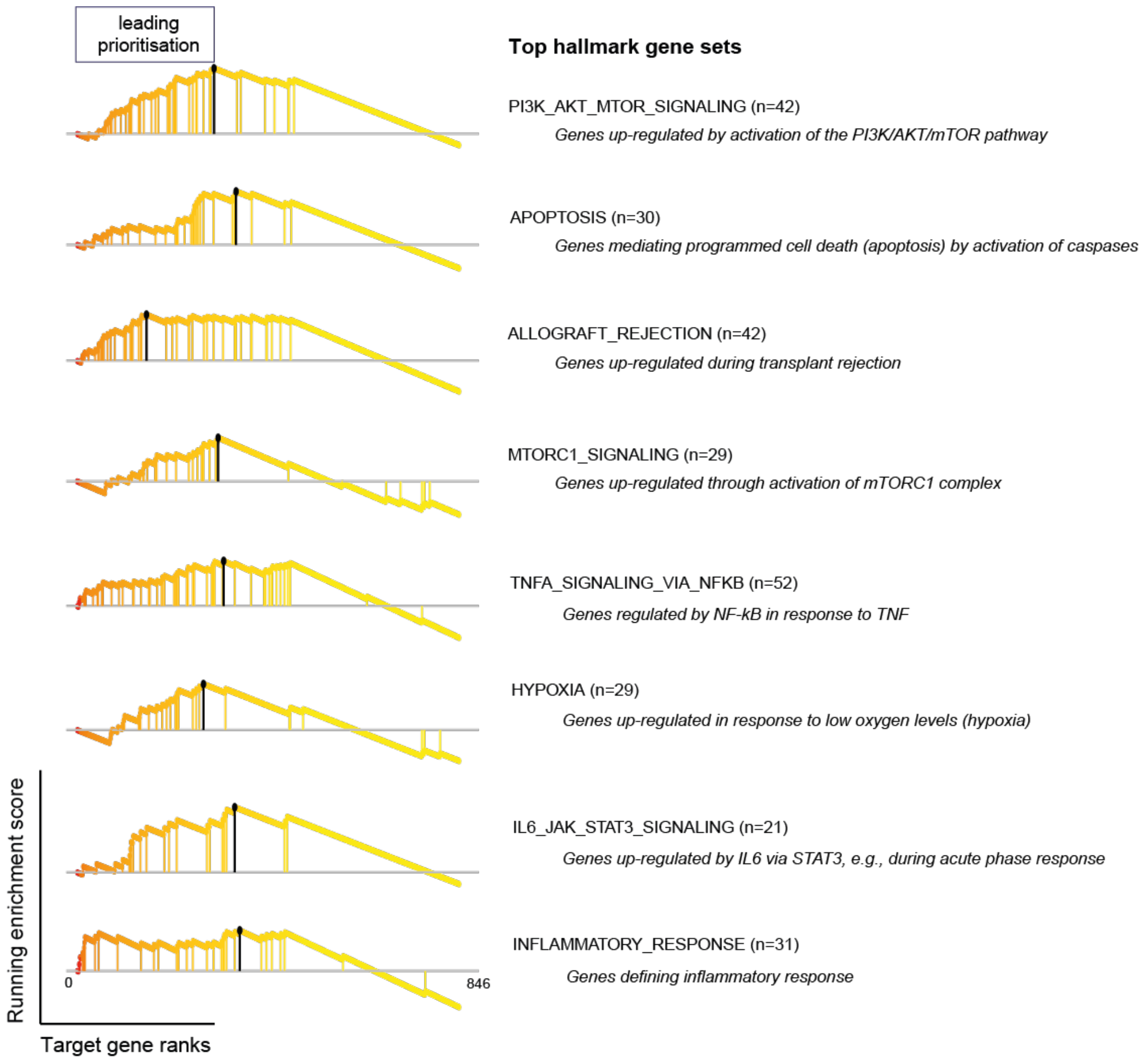

Supplementary Figure 1. Illustration of target set enrichment analysis (TSEA) results, focusing on the most significant (top) hallmark gene sets. The leading prioritisation is defined as the left-most region ahead of the peak (indicated by the dark blue bar).

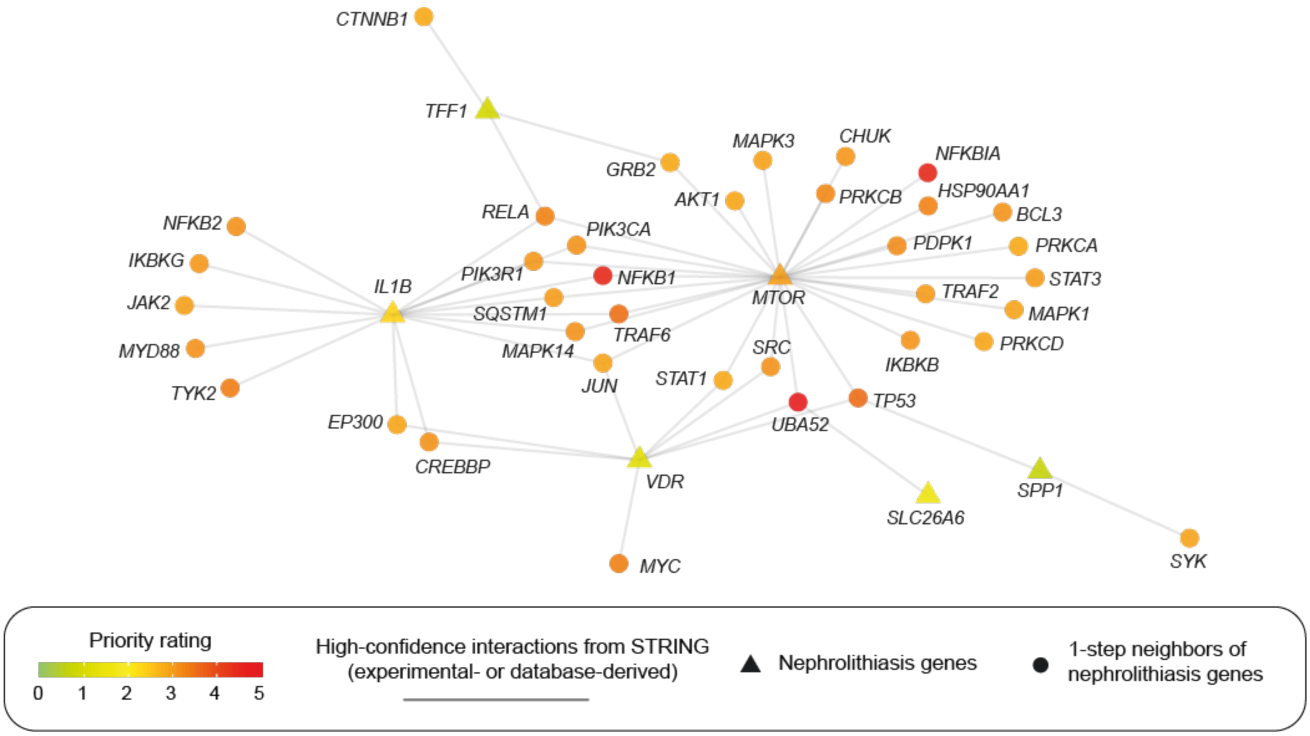

Supplementary Figure 2. Illustration of interaction between nephrolithiasis genes, along with their 1-step (direct) neighbor genes (restricted to the top 1% prioritised genes). Gene nodes are color-coded by priority rating and shaped by whether or not relevant to nephrolithiasis. Gene interactions are sourced from the STRING database, while the relatedness to nephrolithiasis obtained from annotations using Gene Ontology.

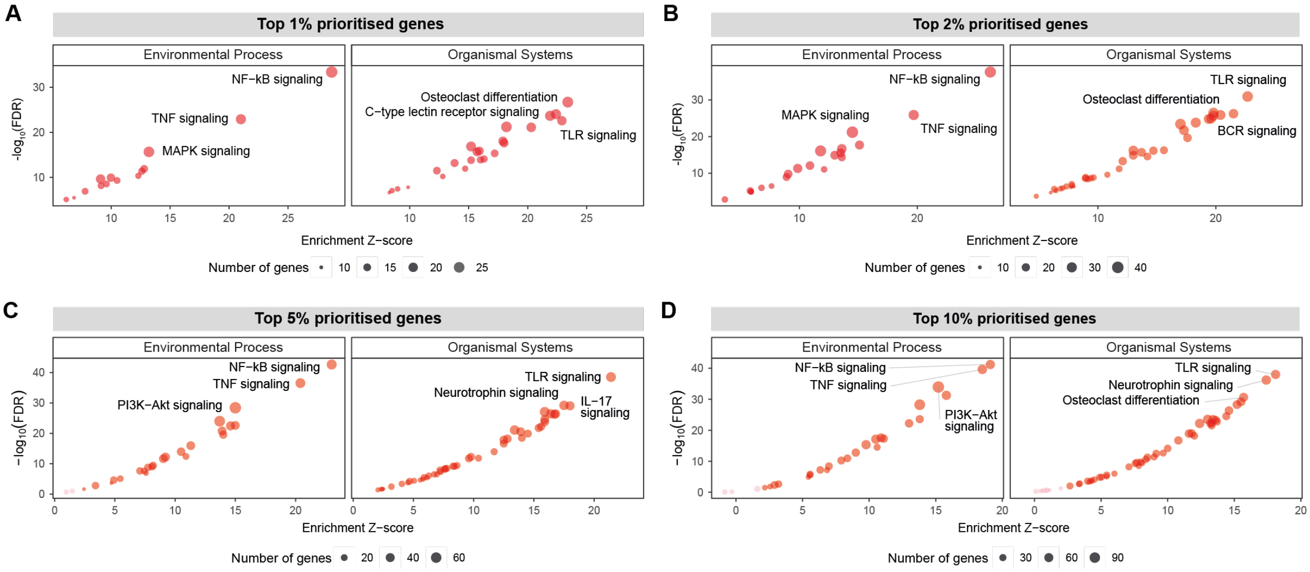

Supplementary Figure 3. Prioritisation of target pathways using the KEGG pathways resource. Scatter plots illustrating prioritised pathways based on (**A**) top 1% prioritised genes, (**B**) top 2% prioritised genes, (**C**) top 5% prioritised genes, and (**D**) top 10% prioritised genes. BCR, B-cell receptor; MAPK, mitogen-activated protein kinase; TLR, toll-like receptor; TNF, tumor necrosis factor.

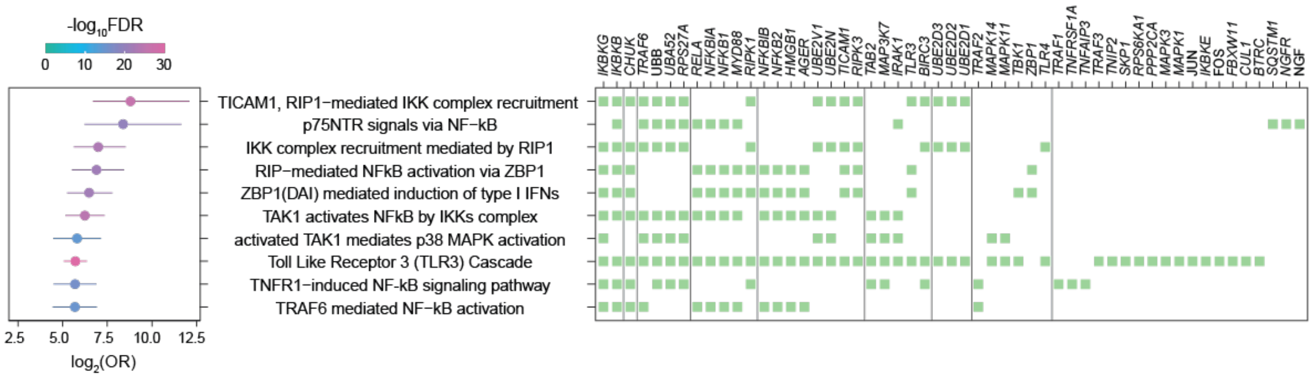

Supplementary Figure 4. Prioritisation of target pathways using the Reactome resource. Forest plots of the top 10 prioritised pathways are shown on the left, with member genes illustrated on the right using a heatmap in which genes are shown at the top and ordered by frequency of occurrence (colored square). OR, odds ratio; IKK, inhibitory kB kinase; IFNs, interferons.

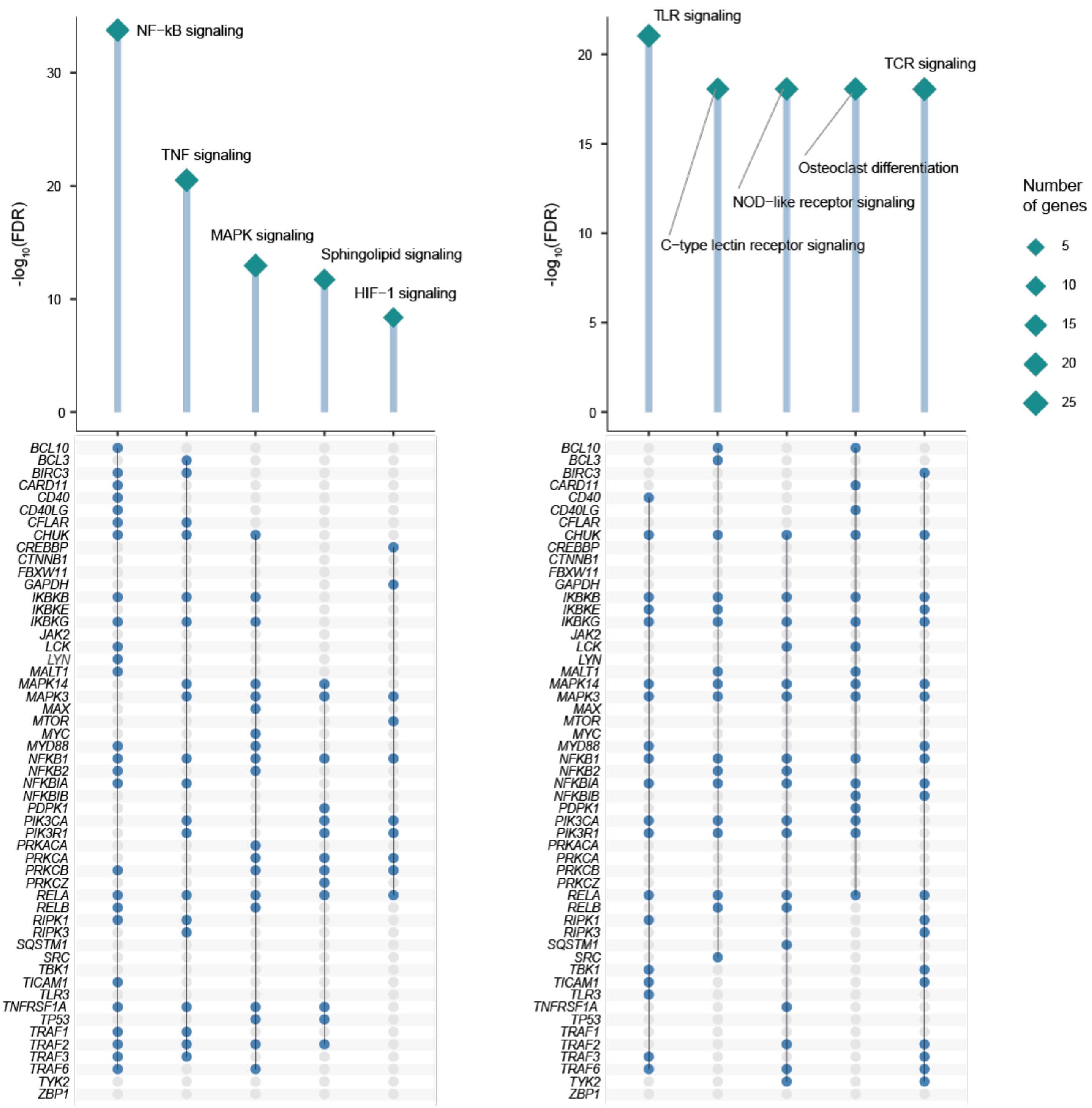

Supplementary Figure 5. The kite-like plot illustrating individual pathways enriched in the crosstalk genes, displayed separately for Environmental Information Processing pathways (left) and Organismal Systems pathways (right). The significance level (FDR) estimated according to Fisher's exact test (one-sided). Each kite sized by the number of member genes (also indicated by blue dots beneath). TCR, T-cell receptor; TLR, toll-like receptor; TNF, tumor necrosis factor.

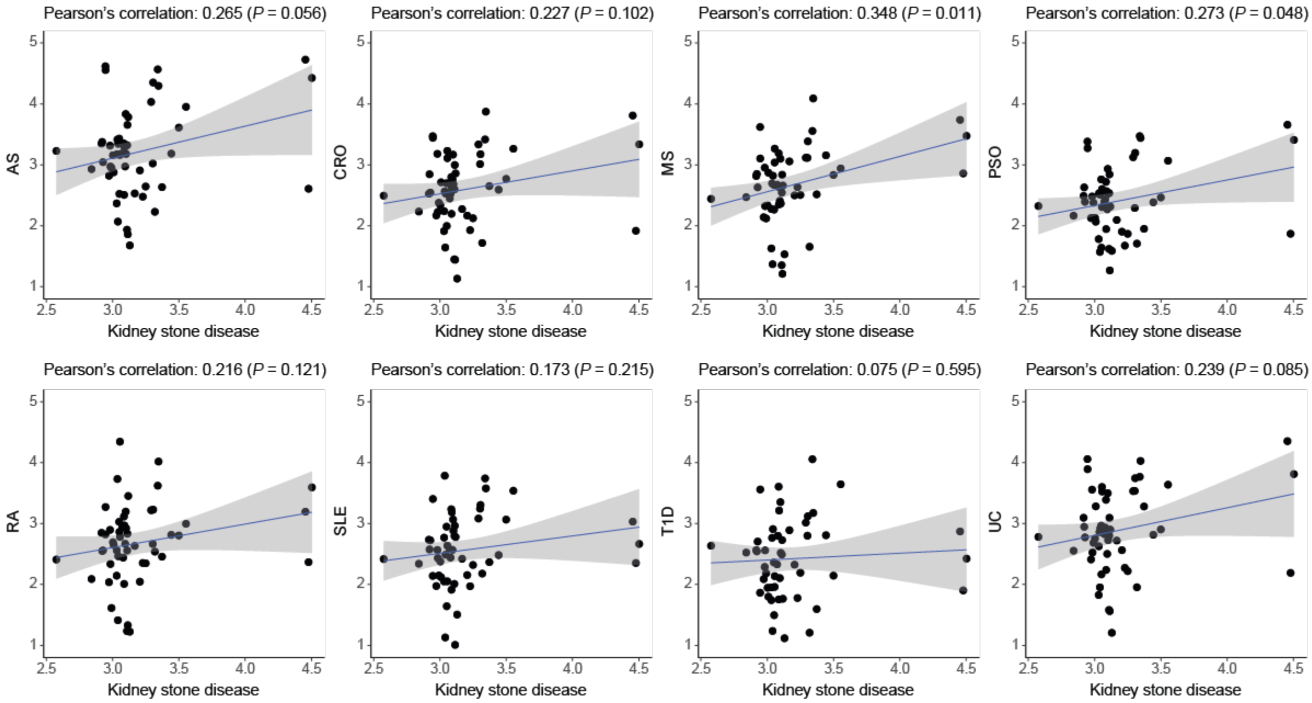

Supplementary Figure 6. Scatter plot for pathway crosstalk genes between priority rating in kidney stone disease (x-axis) and in immune-mediated disease (as indicated in y-axis). The significance level (*P*) for the correlation calculated based on Pearson's test (two sided).

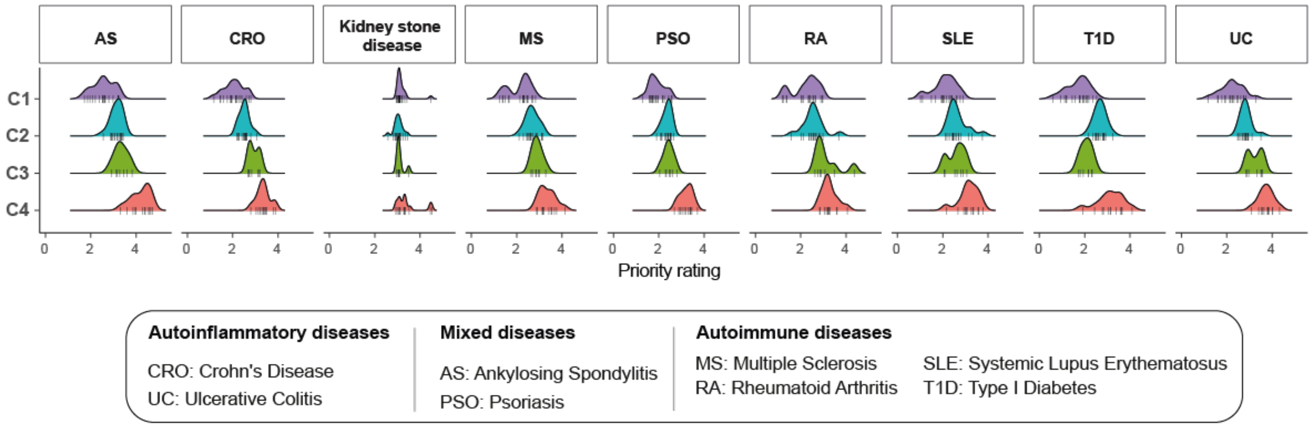

Supplementary Figure 7. Ridge-like plots showing density of priority rating, displayed separately for each of four target clusters per disease. It can be seen that the cluster C4 is highly rated in all diseases, while the cluster C1 highly rated in kidney stone disease only.

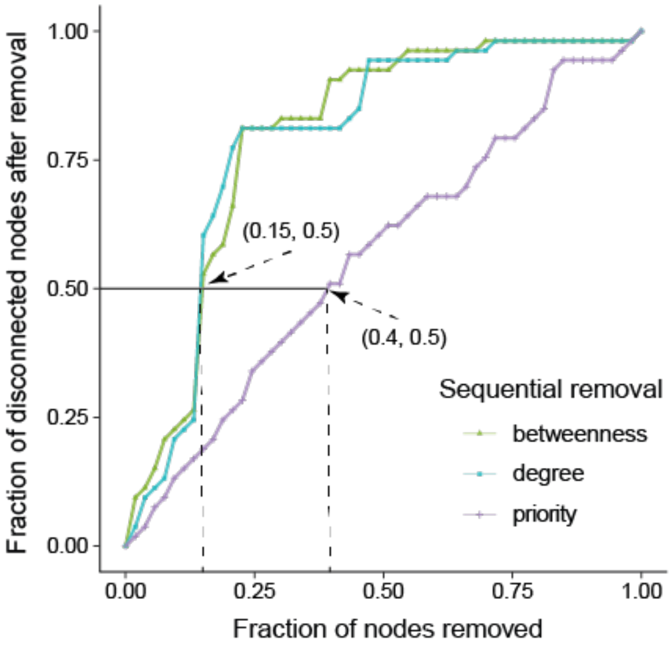

Supplementary Figure 8. Plots showing fraction of nodes removed (x-axis) against fraction of disconnected nodes (y-axis). Each colored curve shows an analysis trajectory along successive node removal, in which nodes are removed sequentially in a decreasing order, either by node centrality (measured by degree or betweenness) or node priority (that is, priority rating). Such successive node removal is known as 'targeted attack'.

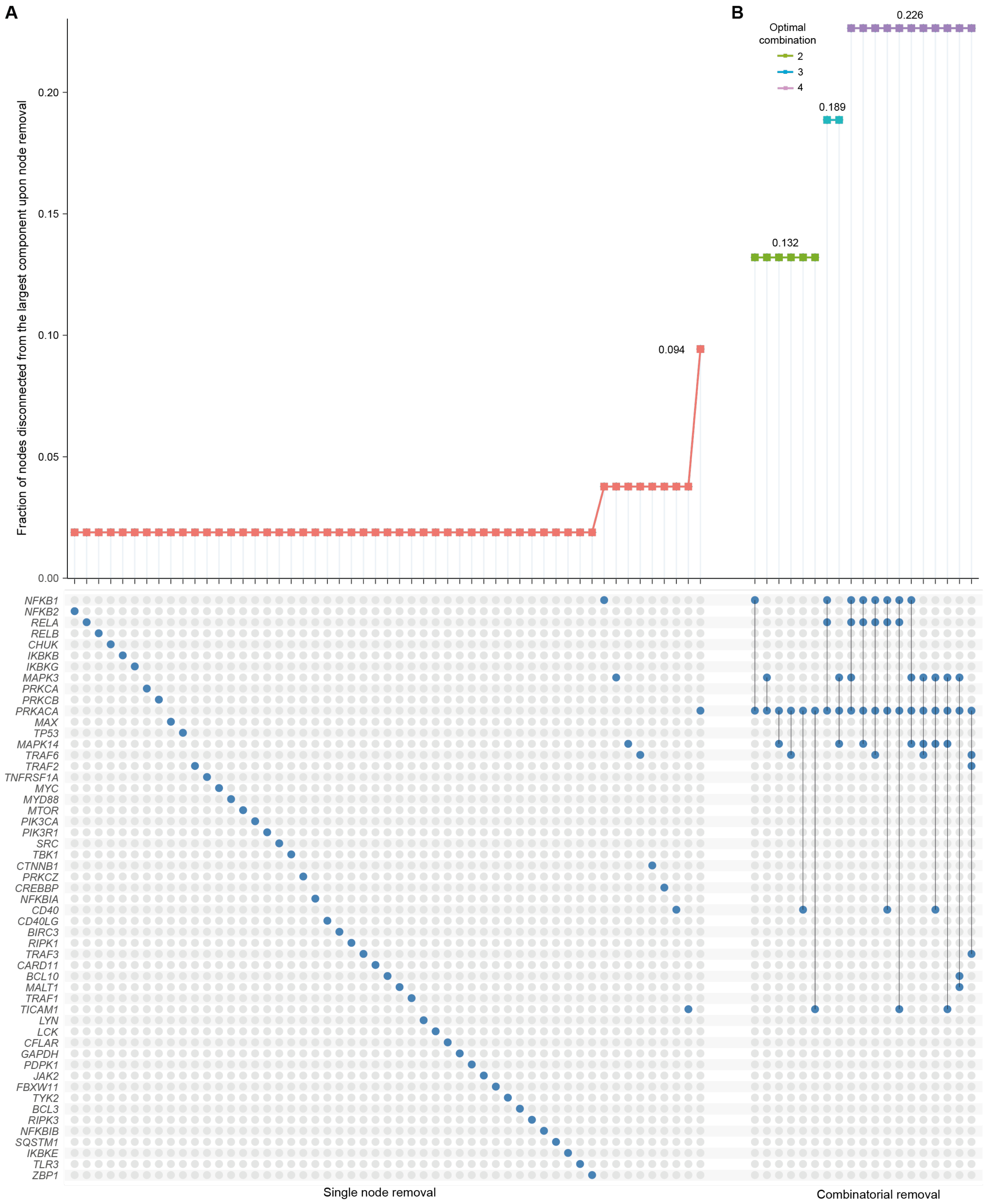

Supplementary Figure 9. Effects of node removal on the crosstalk. Fraction of nodes disconnected from the largest remaining component (y-axis) is plotted against node removal (x-axis). The nodes removed are indicated by blue circles beneath. (**A**) Single node removal. (**B**) Combinatorial removal.
